# Ocular Axial Length and Its Associations in Chinese: The Beijing Eye Study

**DOI:** 10.1371/journal.pone.0043172

**Published:** 2012-08-21

**Authors:** Guo Yin, Ya Xing Wang, Zhi Yun Zheng, Hua Yang, Liang Xu, Jost B. Jonas, the Beijing Eye Study Group

**Affiliations:** 1 Beijing Institute of Ophthalmology, Beijing Tongren Hospital, Capital Medical University, Beijing, China; 2 Yayuncun Clinics, PLA General Armament Department, Beijing, China; 3 Department of Ophthalmology, Medical Faculty Mannheim of the Ruprecht-Karls-University, Heidelberg, Germany; Univeristy of Melbourne, Australia

## Abstract

**Purpose:**

To investigate the normative data of ocular axial length and its associations in Chinese.

**Method:**

The population-based Beijing Eye Study 2011 is a cross-sectional study performed in Greater Beijing. The study included 3468 individuals (1963 (56.6%) women) with a mean age of 64.6±9.8 years (range: 50–93 years). A detailed ophthalmic and medical examination was performed. Axial length was measured by optical low-coherence reflectometry.

**Results:**

Axial length measurements were available for 3159 (91.1%) study participants. Mean axial length was 23.25±1.14 mm (range: 18.96–30.88 mm). In multivariate analysis, axial length was significantly associated with the systemic parameters of higher age (P<0.001), higher body height (P = 0.003), higher level of education (P<0.001) and urban region of habitation (P<0.001), and with the ocular parameters of thicker central cornea (P = 0.001), higher corneal curvature radius (P<0.001), deeper anterior chamber (P<0.001), thicker lens (P<0.001), more myopic refractive error (P<0.001), larger pupil diameter (P = 0.018), and higher best corrected visual acuity (P<0.001). It was additionally and negatively associated with the lens vault (P<0.001). In highly myopic eyes, axial length was significantly associated with lower level of education (P = 0.008), more myopic refractive error (P<0.001), and lower best corrected visual acuity (P = 0.034).

**Conclusions:**

Mean ocular axial length in the older adult population of Greater Beijing (23.25±1.14 mm) was similar to the value measured in other urban populations and was higher than in a rural Central Indian population. The association between axial length and older age may potentially be associated with a survival artifact. The association between axial length and body height agrees with the general association between anthropomorphic measures and eye globe size. The association with the level of education and urban region of habitation confirms with previous studies. In contrast in highly myopic eyes, axial length was negatively associated with educational level and best corrected visual acuity.

## Introduction

Variations in refractive error in older adults with an age of 50 years or more are mostly influenced by variations in axial length and in crystalline lens refractive power, followed by variations in corneal refractive power, and to minor degree by variations in lens thickness and anterior chamber depth [1]. Also, myopia and hyperopia and thus indirectly the axial length are associated with the prevalence of some retinal diseases, such as age-related macular degeneration, and with the prevalence of various forms of glaucoma [2]. Axial length is therefore one of the basic anatomic parameters in ophthalmology and a major variable for the optical quality of the image on the retina. Despite of its importance, however, relatively few studies were focused on axial length in population-based studies. Investigations which among others are included the list of these studies were performed for study populations from Singapore, South and Central India, Alaska, Mongolia, California, Myanmar, Wisconsin, England, and other regions [3]–[15]. In addition, although China is the country with the largest population worldwide, there has been only one population-based study, the Liwan study from South China, on axial length with no data measured in a population-based study for North China [16]. North China and South China, located at a distance of more than 4500 km from each other, show however pronounced differences in climate, geography, living circumstances and nutrition, to mention only few parameters. It was therefore the purpose of our study to obtain normative data of axial length and to examine associations between axial length and other ophthalmic parameters (such as corneal curvature) and systemic parameters (such as general anthropomorphic measurements and socioeconomic data) in a relatively large population from North China.

## Methods

### Ethics Statement

The Medical Ethics Committee of the Beijing Tongren Hospital approved the study protocol and all participants gave informed written consent, according to the Declaration of Helsinki.

The Beijing Eye Study 2011 is a population-based cross-sectional study in Northern China. It was carried out in 5 communities in the urban district of Haidian in the North of Central Beijing and in 3 communities in the village area of Yufa of the Daxing District south of Beijing. The only eligibility criterion for inclusion into the study was an age of 50+ years. The reason to perform the study in a rural area and in an urban area was that both areas differed markedly in the level of education, access to medical care, mobility, frequency of hereditable diseases, and way of life. In the rural areas, eye care services and a referral system to ophthalmologists was markedly less developed than in the urban areas, where eye care was at a relatively high standard with some communities supplying free ophthalmic examinations. The rural region of Yufa of the Daxing District and the urban region of Haidian were chosen since they were considered to be typical for a rural region and an urban region, respectively, in the Greater Beijing region. In 2011, the 8 communities had a total population of 4403 individuals aged 50 years or older. In total, 3468 individuals (1963 (56.6%) women) participated in the eye examination, corresponding to an overall response rate of 78.8%. More than 99.5% of the study population was ethnically Han Chinese. The study was divided into a rural part (1633 (47.1%) subjects; 943 (57.7%) women) and an urban part (1835 (52.9%) subjects; 1020 (55.6%) women). The mean age was 64.6±9.8 years (median, 64 years; range, 50–93 years). The Beijing Eye Study 2011 was performed in the same seven settlements as the previous Beijing Eye Studies 2001 and 2006, plus in one additional community in the urban region. It included all participants from the previous two investigations [17], [18], who participated in a follow-up manner in the survey from 2011, and it added in a cross-sectional manner those subjects who fulfilled the eligibility criterion of an age of 50+ years, who lived in the study region and who had not participated in the previous surveys.

All examinations were carried out in the communities, either in schoolhouses or in community houses (e.g. general assembly houses, auditoria, or large office rooms for the village). All study participants underwent a detailed interview by trained health staff with standardized questions on their family status, level of education, income, quality of life, psychic depression, physical activity, and known major systemic diseases. The body height was determined in a standardized manner with the shoes routinely removed. The subjects were asked to stand upright as much as possible and with the head raised upright as much as possible. We used a stadiometer as measuring instrument. We did not take into account nor corrected age-related reductions in height of subjects who reportedly were taller during their middle-age. Body height and weight and the circumference of the waist and hip were recorded. The ophthalmic examination included measurement of presenting visual acuity and uncorrected visual acuity. If uncorrected visual acuity was lower than 1.0 (or logMAR (negative decadal logarithm of the minimal angle of resolution) higher than 0.00), best corrected visual acuity was assessed after automatic refractometry (Auto Refractometer AR-610, Nidek Co., Ltd, Tokyo, Japan) was carried out. Intraocular pressure was measured by pneumotonometry. A slit lamp examination carried out by an experienced ophthalmologist assessed lid abnormalities, Meibomian gland dysfunction, corneal disorders, and peripheral anterior chamber depth using van Herick's method. Gonioscopy was routinely performed for all study participants. Using optical low-coherence reflectometry (Lensstar 900® Optical Biometer, Haag-Streit, 3098 Koeniz, Switzerland), biometry of the right eyes was performed for measurement of the anterior corneal curvature, central corneal thickness, anterior chamber depth, lens thickness and axial length. The examination was performed by experienced clinical technicians. Five measurements were performed, and the mean value was taken for further statistical analysis. The pupil was dilated using tropicamide 1% eye drops (Mydrin; Santen; Japan). If after the instillation of the first drop of tropicamide the pupil was not sufficiently dilated, the instillation was repeated, until the pupil diameter was at least 6 mm. A second slit lamp assisted biomicroscopy searched for pseudoexfoliation syndrome. Digital photographs of the cornea and lens and retro-illuminated photographs of the lens were taken using the Neitz CT-R camera (Neitz Instruments Co., Tokyo, Japan). Monoscopic photographs of the macula and optic disc were taken using a fundus camera (Type CR6-45 NM, Canon Inc. U.S.A.). For a subgroup of subjects, the anterior segment of the right eyes was measured by slit-lamp adapted optical coherence tomography (OCT) (Heidelberg Engineering Co., Dossenheim, Germany) (scan size: max.15 mm; Scan depth: 7 mm; lateral optical resolution capacity: 20–100 µm; axial optical resolution capacity: <25 µm; diode laser: 1310 nm) in a previous survey in 2006 [18]. We measured the anterior chamber depth and the anterior chamber angle in the temporal region and the nasal region using a software program supplied with the device. The lens vault was defined as the perpendicular distance between the anterior pole of the crystalline lens and the horizontal line joining the two scleral spurs on the horizontal anterior segment OCT scans. In addition, the central corneal thickness was determined. This present study was focused on axial length and its associations with ocular and general parameters. Only those subjects with axial measurements were included in the study described herein.

Statistical analysis was performed using a commercially available statistical software package (SPSS for Windows, version 20.0, IBM-SPSS, Chicago, IL). In a first step, we determined the mean value (presented as mean ± standard deviation) of the main outcome parameter. The Gaussian distribution of the parameters was tested using the Kolmogorov-Smirnov test. In a second step, we performed univariate analyses of the associations between axial length and other ocular and systemic parameters. As the third step, we carried out multivariate regression analyses with the axial length as the dependent parameter and as independent parameters all of those that were significantly associated with axial length in the univariate analyses. In a fourth step, we removed from the multivariate analysis, step-by-step, those parameters that were no longer significantly associated with axial length, starting with those parameters that showed the least probable association (highest *P*-value). In a fifth step, we excluded all highly myopic eyes with a myopic refractive error of more than -8 diopters and repeated the statistical analysis. Finally, only highly myopic eyes were included into the statistical analysis. Only one randomly selected eye from each subject was used for statistical analysis. For the assessment of the association between axial length and refractive error, pseudophakic and aphakic eyes were excluded.

## Results

Axial length measurements were available for 3159 (91.1%) study participants (1787 (56.6%) women). The group of subjects without axial length measurements as compared with the group of subjects with axial length measurements was significantly (*P*<0.001) older and did not vary significantly in gender (*P* = 1.0). The mean age in the study population was 64.1±9.8 years (median, 63 years; range, 50–93 years), and the mean refractive error was −0.18±2.08 diopters (median, +0.50 diopters; range, −25.0 to +7.00 diopters). Mean central corneal thickness was 532±33 µm, mean anterior chamber depth was 2.49±0.50 mm, mean lens thickness was 4.56±0.34 mm, mean flattest keratometric reading was 7.69±0.26 mm, and mean steepest keratometric reading was 7.55±0.26 mm. Intraocular pressure averaged 14.5±2.7 mmHg. Anterior segment optical coherence tomography (OCT) measurements were available for 1866 participants (1090 (58.4%) women) with a mean age of 64.0±9.6 years (range: 50–93 years) and a mean refractive error of was −0.15±2.15 diopters (median, +0.25 diopters; range, −22.0 to +7.00 diopters). The subgroup of subjects with OCT measurements and those without did not differ significantly in age (*P* = 0.28) and refractive error (*P* = 0.42).

**Figure 1:**
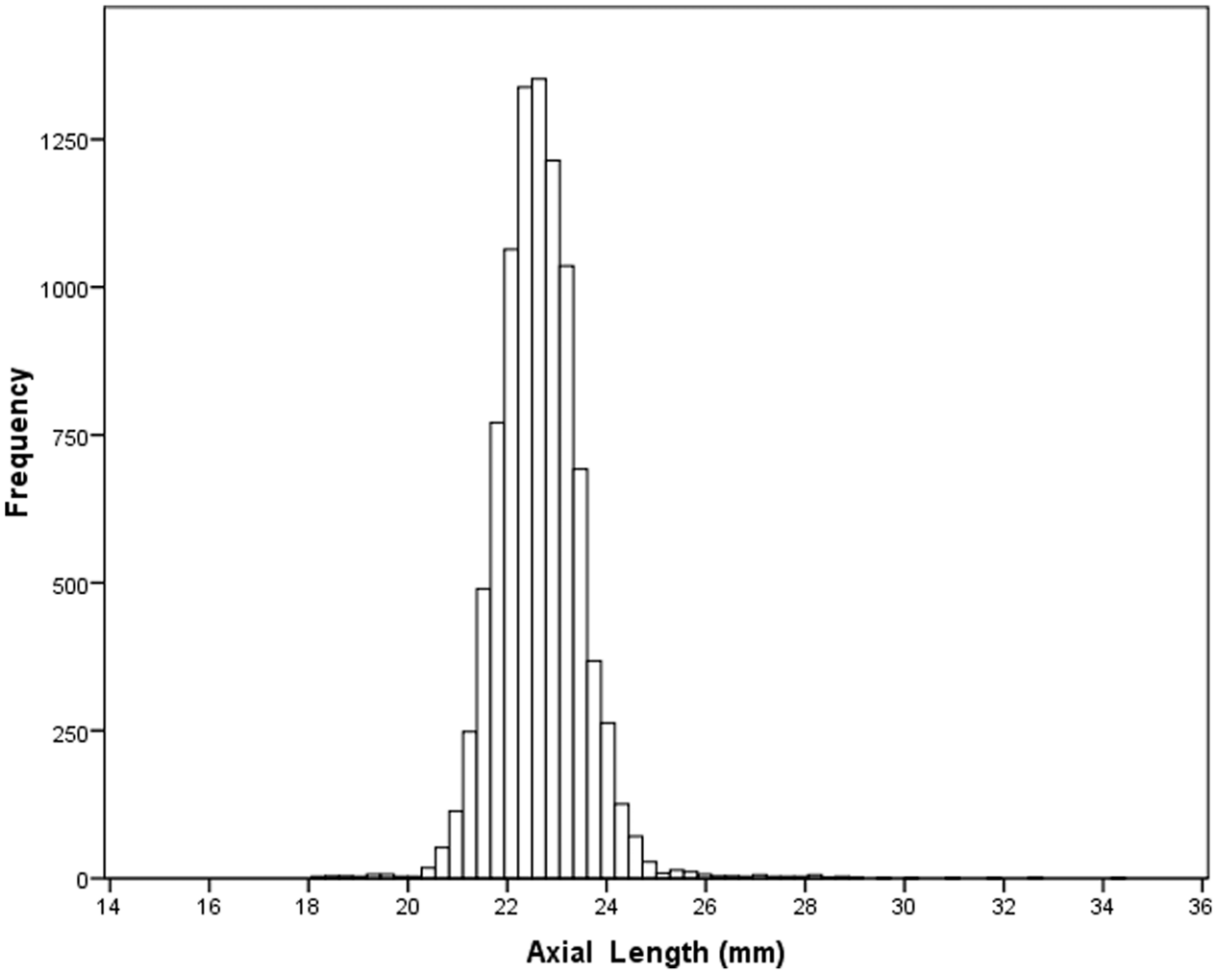
Histogram showing distribution of the ocular axial length in the Beijing Eye Study 2011.

Mean axial length was 23.25±1.14 mm (median, 23.14 mm; range, 18.96 mm to 30.88 mm; kurtosis: 5.49; skew: 1.41) (Fig 1). It was not normally distributed (Kolmogorov-Smirnov test; *P*<0.001). In univariate analyses, axial length was significantly associated with the systemic parameters of male gender, greater body height, body weight, body mass index, higher level of education and urban region of habitation; and with the ocular parameters of a thicker and flatter cornea, a larger corneal diameter, a deeper anterior chamber, a thinner lens, a more myopic refractive error, and lower best corrected visual acuity (Table 1). It was not significantly associated with intraocular pressure (*P* = 0.40). Axial length was significantly (*P*<0.001) and positively associated with the anterior segment OCT parameters of anterior chamber depth and volume, posterior corneal curvature, nasal and temporal anterior chamber angle (ACA) at 500 µm and 750 µm from the scleral spur, nasal and temporal anterior chamber opening distance (AOD) at 500 µm and 750 µm from the scleral spur, and nasal and temporal trabecular-iris-space area (TISA) at 500 µm and 750 µm from the scleral spur, and negatively with the lens vault.

**Table 1:** Univariate Analysis of Associations between Axial Length and Systemic and Ocular Parameters in the Beijing Eye Study 2011.

	*P* Value	Correlation Coefficient	Steepness of the Regression Line	95% CI of Steepness of Regression Line
Systemic Parameters				
Age (Years)	<0.001	0.09	0.01	0.006, 0.014
Gender	<0.001	−0.23	−0.52	−0.61, −0.45
Body height (cm)	<0.001	0.29	0.04	0.04, 0.05
Body weight (kg)	<0.001	0.09	0.01	0.005, 0.012
Body mass index	<0.001	−0.10	−0.03	−0.04,−0.02
Level of education	<0.001	0.26	0.28	0.24, 0.32
Rural/Urban Region	<0.001	0.24	0.54	0.46, 0.62
Ocular Parameters				
Central Corneal Thickness ( µm)	<0.001	0.11	0.004	0.003, 0.005
Anterior Corneal Curvature (mm)				
Flattest Meridian	<0.001	0.52	2.27	2.14, 2.40
Steepest Meridian	<0.001	0.49	2.12	1.99, 2.26
Corneal diameter (mm)	0.001	0.06	0.07	0.03, 0.11
Anterior Chamber depth (mm)	<0.001	0.34	0.77	0.69, 0.84
Lens thickness (mm)	<0.001	−0.11	−0.37	−0.49, −0.25
Refractive error (Diopters): Spherical error	<0.001	−0.60	−0.35	−0.36, −0.33
Cylindrical error (absolute)	0.046	0.04	0.06	0.001, 0.11
Spherical equivalent	<0.001	−0.62	−0.34	−0.35, −0.32
Intraocular pressure (mmHg)	0.396	0.02	0.006	−0.01, 0.02
Pupil diameter (mm)	<0.001	0.21	0.30	0.25, 0.35
Pupil distance	<0.001	0.18	0.05	0.04, 0.05
BCVA (logMAR)	<0.001	0.08	0.41	0.18, 0.63

*P*-Value = statistical significance of the association

CI: = confidence interval

BCVA: = best corrected visual acuity

logMAR: = logarithmic value of the minimal angle of resolution

In the multivariate linear regression analysis, axial length remained to be significantly associated with the systemic parameters of higher age, higher body height, higher level of education and urban region of habitation, and with the ocular parameters of thicker central cornea, higher corneal curvature radius, deeper anterior chamber, thicker lens, more myopic refractive error, larger pupil diameter, and higher best corrected visual acuity (Table 2). If parameters, that were no longer significantly associated with axial length, were removed step-by-step from the multivariate analysis, the same results were obtained (Table 3). In the subgroup of study participants with anterior segment OCT measurements, all OCT parameters, except of lens vault (*P*<0.001; standardized coefficient Beta: −0.09) were no longer significantly associated with axial length.

**Table 2:** Multivariate Analysis of Associations between Axial Length and Systemic and Ocular Parameters in the Beijing Eye Study 2011.

	*P-*Value	Regression Coefficient (non-standard Beta)	95% CI of Un-Standardized Regression Coefficient	Standardized Regression Coefficient
Systemic Parameters				
Age (Years)	<0.001	0.02	0.02, 0.03	0.20
Gender	0.32	−0.02	−0.07, 0.02	−0.01
Body height (cm)	0.01	0.004	0.001, 0.007	0.03
Body weight (kg)	0.86	0.000	−0.002, 0.002	0.002
Level of education	<0.001	0.04	0.02, 0.03	0.04
Rural/Urban Region	<0.001	0.12	0.07, 0.16	0.05
Ocular Parameters				
Central corneal thickness ( µm)	<0.001	0.001	0.001, 0.002	0.03
Anterior corneal curvature, flat meridian (mm)	<0.001	1.04	0.84, 1.24	0.24
Anterior corneal curvature, steep meridian (mm)	<0.001	1.06	0.86, 1.26	0.25
Corneal Diameter (mm)	0.25	−0.01	−0.028, 0.007	−0.01
Pupil Diameter (mm)	0.02	0.03	0.005, 0.048	0.02
Pupil distance (mm)	0.040	−0.004	−0.008, 0.000	−0.02
Anterior chamber depth (mm)	<0.001	1.20	1.14, 1.27	0.37
Lens thickness (mm)	<0.001	0.20	0.14, 0.26	0.06
Refractive error (Diopters):Spherical Equivalent	<0.001	−0.29	−0.33, −0.24	−0.51
Spherical error	0.08	−0.04	−0.09, 0.004	−0.07
Cylindrical error	0.59	−0.01	−0.04, 0.02	−0.006
BCVA (logMAR)	<0.001	−0.76	−0.94, −0.59	−0.08

*P*-value = statistical significance of the association

CI = confidence interval

BCVA = best corrected visual acuity

logMAR = logarithmic value of the minimal angle of resolution

**Table 3:** Multivariate Analysis of Associations between Ocular Length and Systemic and Ocular Parameters as Independent Parameters that Remained Significantly Associated with Axial Length after Stepwise Exclusion of Parameters.

	*P*- Value	Regression Coefficient (un-standardized Beta)	95% CI of Un-Standardized Regression Coefficient	Standardized Regression Coefficient	Variance Inflation Factor
Systemic Parameters					
Age (Years)	<0.001	0.02	0.02, 0.04	0.20	1.84
Body height (cm)	<0.001	0.006	0.004, 0.009	0.05	1.38
Level of education	<0.001	0.05	0.03, 0.07	0.04	1.64
Rural/Urban Region	<0.001	0.11	0.07, 0.15	0.05	1.67
Ocular Parameters					
Central corneal thickness ( µm)	<0.001	0.001	0.001, 0.002	0.03	1.05
Anterior Corneal Curvature (mm), flat meridian	<0.001	1.05	0.88, 1.22	0.24	7.23
Anterior Corneal Curvature (mm), steep meridian	<0.001	1.06	0.90, 1.23	0.25	7.26
Pupil diameter (mm)	0.01	0.03	0.01, 0.05	0.02	1.14
Pupil distance (mm)	0.052	−0.004	−0.01, 0.000	−0.02	1.16
Anterior chamber depth (mm)	<0.001	1.13	1.07, 1.19	0.35	1.67
Lens thickness (mm)	<0.001	0.14	0.08, 0.20	0.04	1.56
Refractive error (Diopters)	<0.001	−0.32	−0.33, −0.32	−0.58	1.36
BCVA (logMAR)	<0.001	−0.79	−0.96, −0.61	−0.08	1.72

*P* value = statistical significance of the association

CI = confidence interval

BCVA = best corrected visual acuity

logMAR = logarithmic value of the minimal angle of resolution

If highly myopic eyes (n = 34 subjects) with an axial length of ≥26.5 mm were excluded from the analysis, the results remained essentially the same (Table 4). Within this relatively small group of subjects with high myopia (axial length ≥26.5 mm), increasing axial length (multivariate analysis) was significantly associated with lower level of education (*P* = 0.008; standardized regression coefficient Beta =  −0.37), more myopic spherical refractive power (*P*<0.001; Beta =  −0.82), and lower best corrected visual acuity (*P* = 0.034; Beta =  −0.28).

**Table 4:** Multivariate Analysis of Associations between Ocular Axial Length and Systemic and Ocular Parameters in the Beijing Eye Study 2011. Highly Myopic Eyes with a refractive error of more than −8 diopters were excluded.

	*P*- Value	Regression Coefficient (un-standardized Beta)	95% CI of Un-Standardized Regression Coefficient	Standardized Regression Coefficient	Variance Inflation Factor
Systemic Parameters					
Age (Years)	<0.001	0.02	0.02, 0.03	0.23	1.89
Body height (cm)	<0.001	0.006	0.004, 0.008	0.05	1.38
Level of education	<0.001	0.06	0.04, 0.07	0.06	1.64
Rural/Urban Region	<0.001	0.11	0.07, 0.14	0.05	1.67
Ocular Parameters					
Central corneal thickness ( µm)	<0.001	0.001	0.001, 0.002	0.04	1.05
Anterior Corneal Curvature (mm) flat	<0.001	0.96	0.81, 1.12	0.25	7.26
Anterior Corneal Curvature (mm) steep	<0.001	1.12	0.96, 1.27	0.29	7.29
Pupil diameter (mm)	0.002	0.03	0.01, 0.05	0.03	1.15
Pupil distance (mm)	0.069	−0.003	−0.007, 0	−0.02	1.16
Anterior chamber depth (mm)	<0.001	1.13	1.08, 1.19	0.39	1.65
Lens thickness (mm)	<0.001	0.12	0.06, 0.18	0.04	1.55
Refractive error (Diopters)	<0.001	−0.30	−0.31, −0.29	−0.48	1.26
BCVA (logMAR)	<0.001	−0.81	−0.98, −0.63	−0.09	1.65

*P* value = statistical significance of the association

CI = confidence interval

BCVA = best corrected visual acuity

logMAR = logarithmic value of the minimal angle of resolution

## Discussion

In an older adult population of Greater Beijing, the mean ocular axial length was found to be 23.3±1.1 mm (median: 23.14 mm) ranging from 18.96 mm to 30.88. In multivariate analysis, axial length was significantly associated with the systemic parameters of higher age, higher body height, higher level of education and urban region of habitation, and with the ocular parameters of thicker central cornea, higher corneal curvature radius, deeper anterior chamber, thicker lens, less lens vault, more myopic refractive error, larger pupil diameter, and higher best corrected visual acuity. In contrast, in highly myopic eyes, axial length was significantly associated with lower level of education and lower best corrected visual acuity.

The mean axial length was considerably larger in our study population than in a rural Central Indian population (population-based Central Indian Eye and Medical Study: mean age: 49.4±13.4 years; method: ultrasonic biometry; axial length: 22.6±0.91 mm) and in a South Indian population (population-based Chennai Glaucoma Prevalence Study: mean age: 50.0±10.0 years; method: ultrasonic biometry; axial length: 22.8±0.8 mm) [5], [14]. It was almost identical to the axial length measured in South China in the population-based Liwan Study (mean age: 64.4±9.6 years; method: ultrasonic biometry; axial length: 23.11 mm) [16] and it was similar to the axial length measured in a rural Mongolian population (age: 40+ years; method: ultrasonic biometry; axial length: 23.1±1.2 mm) [7], in the population of the Tanjong Pagar Study in Singaporean Chinese aged 40+ years (method: ultrasonic biometry; axial length: 23.2±1.2 mm) [3], in the population of the Los Angeles Latino Eye Study (mean age: 54.2 years; method: ultrasonic biometry; axial length: 23.4±1.1 mm) [8]. In the Beaver Dam Eye Study (age: 58–100 years; method: partial coherence laser interferometry; mean axial length: 23.69±1.16 mm) and in the EPIC-Norfalk study from England (2519 subjects aged 48 to 88 years; method: partial coherence laser interferometry; mean axial length: 23.80±1.16 mm (men; 23.29±1.15 mm (women)) ) mean axial length was slightly longer than in our study [13], [15].

With respect to the associations between axial length and systemic and ocular parameters, our study agrees with previous investigations. In the Central Indian Eye and Medical Study as in our study, axial length increased with higher age, taller body height, higher level of education, lower corneal refractive power, deeper anterior chamber, thicker lens, more myopic spherical and cylindrical refractive power, and lower best corrected visual acuity [14]. As in our study, axial length increased with age in the Mongolian study [7]. Our study agrees with the Tanjong Pagar Study in that axial length was associated with body height, higher socioeconomic background, and deeper anterior chambers [19], [20]. In contrast to our study, however, axial length decreased with increasing age in the Tanjong Pagar Study, and it was not associated with lens thickness. Also, in the Tanjong Pagar Study, women had shorter globes after adjusting for age, and heavier people tended to have shorter eyes, while in our study subjects axial length was not significantly (*P* = 0.78) associated with body mass index after adjustment for the parameters mentioned above. In contrast to our study, the Los Angeles Latino Eye Study did not reveal an association between axial length and age, and in contrast to our study, women had shorter eyes than did men [8]. In the Beaver Dam Eye Study in contrast to our study, axial length decreased with higher age and axial length was greater in men than in women [13]. As in our study, axial length was associated with taller height and with higher education.

The association between axial length and a higher level of education agrees with all previously mentioned investigations on other populations [13], [14], [19]. In a parallel manner, the studies including the present investigation agree on the association between increasing axial length and urban region of habitation. Our finding that axial length increased with age was in contrast to most other population-based studies except of the Central India Eye and Medical Study [14].The reasons for this discrepancy between the studies have remained unclear so far. It cannot be explained by a markedly rural or markedly urban character of our study population, since the same finding was obtained in the rural population from the Central India Eye and Medical Study. The association between axial length and body height reflects a general tendency that taller subjects have larger eyes, as has already been shown in other population-based studies such as the Singaporean Tanjong Pagar Study [20], the Icelandic Reykjavik Eye Study [21], the Burmese Meiktila Eye Study [10], and the Central India Eye and Medical Study [14]. Considering the associations between axial length, anterior chamber depth and body height as found in our and other studies, and taking a shallow and small anterior chamber (and indirectly a short axial length) as surrogate for the risk of primary angle-closure glaucoma [22], one may assume that short subjects may have a higher risk to develop primary angle-closure glaucoma. That assumption agrees with a finding from the Beijing Eye Study, in which the prevalence of primary angle-closure glaucoma was associated with shorter body stature [23].

A new finding of our study was that in the highly myopic study participants, as in contrast to the medium myopic or emmetropic study participants, axial length was reversely associated with the level of education. It suggests that high myopia, defined as an axial length of approximately ≥26.5 mm (or a myopic refractive error of approximately more than -8 diopters), differed from other groups of axial length and that it may thus be considered as a separate group in the analysis of population-based studies. The exceptional position of the highly myopic subjects as compared to the remaining subjects was also demonstrated in other studies in which highly myopic eyes as compared to non-highly myopic eyes showed specific features in the morphology of the optic nerve head, the overall anatomy of the globe, and the susceptibility for diseases such as glaucomatous optic neuropathy [24]–[26].

Potential limitations of our study should be mentioned. First, a major concern in any prevalence study is nonparticipation. The Beijing Eye Study 2011 had a reasonable response rate of 78.8%, however, differences between participants and non-participants could have led to a selection artifact. Since the group of subjects without axial length measurements as compared to the group of subjects with axial length measurements was significantly older, one may argue that the non-participation of a part of the elderly eligible study population may have influenced the results of the investigation. Second, another limitation of the study may be the question how representative the rural region and the urban regions of Greater Beijing were for the whole country. To obtain data from a purely rural region in China, far from any major metropolitan region, the recent Handan Study may be more appropriate than the Beijing Eye Study [27]. The Handan Study was a population-based, cross-sectional study on 6830 Chinese aged 30+ years from villages about 500 km South of Beijing. Third, the comparison of mean axial length values across studies may not be accurate, as different studies used different measurement methods. Fourth, our study population was younger than the majority of the populations of the other population-based investigations what may limit the comparability of the results between the studies. Fifth, the conclusion that longer axial length was associated with a lower level of education among the highly myopic subjects was based on a relatively small group of 34 highly myopic subjects.

In conclusion, in the urban and rural population of Greater Beijing, the mean ocular axial length was 23.25±1.14 mm. This value was similar to values in other urban populations, and it was larger than that of markedly rural populations. Axial length was associated with the systemic parameters of increased age, taller body height, higher level of education and urban region of habitation; and with the ocular parameters of thicker central cornea, higher corneal curvature radius, deeper anterior chamber, thicker lens, lower lens vault, more myopic refractive error, and higher best corrected visual acuity. In contrast in highly myopic eyes axial length was negatively associated with educational level and best corrected visual acuity.
